# Draft Genome Sequence of “*Paramesorhizobium deserti*” A-3-E^T^, a Strain Highly Resistant to Diverse β-Lactam Antibiotics

**DOI:** 10.1128/genomeA.00311-16

**Published:** 2016-04-28

**Authors:** Ruichen Lv, Xianwei Yang, Nan Fang, Yuqin Song, Xuesong Luo, Jingyu Guo, Fang Peng, Ruifu Yang, Yujun Cui, Chengxiang Fang, Yajun Song

**Affiliations:** a State Key Laboratory of Pathogen and Biosecurity, Beijing Institute of Microbiology and Epidemiology, Beijing, China; b Key Laboratory of Dairy Biotechnology and Engineering, Ministry of Education, Inner Mongolia Agricultural University, Hohhot, Inner Mongolia, China; c College of Life Sciences, Wuhan University, Wuhan, China; d State Key Laboratory of Agricultural Microbiology and College of Resources and Environment, Huazhong Agricultural University, Wuhan, China

## Abstract

Here, we report the draft genome sequence of “*Paramesorhizobium deserti*” A-3-E^T^, a strain isolated from the Taklimakan Desert of Xinjiang, China, which is resistant to multiple β-lactam antibiotics and other antibiotics (kanamycin, erythromycin, streptomycin, etc.) as well.

## GENOME ANNOUNCEMENT

The rapid emergence and spreading of antibiotic resistant bacteria have become a major public health concern all over the world. With the selection of antibiotics, spontaneous mutations conferring resistances will be fixed in the clinical bacterial population. On the other hand, acquisition of resistance genes from environmental bacteria via horizontal gene transfer (HGT) also plays a vital role in rendering antibiotic resistance (1, 2). The antibiotic resistomes of soil microorganisms have proven to be major reservoirs for genes resistant to pathogenic bacteria via HGT (3, 4).

During field surveys on the bacterial diversity in the Taklimakan Desert of Xinjiang, China, 10 novel β-lactam antibiotic resistant strains were isolated. One of these strains, A-3-E^T^, grows well in media containing 8,000 mg/L of ampicillin, 1,000 mg/L of cefazolin, or 500 mg/L of cefotaxime, and it also shows resistance to other antibiotics (kanamycin, erythromycin, streptomycin, polymyxin B, etc.). Systematic polyphasic researches revealed that strain A-3-E^T^ represents a novel genus and species of *Proteobacteria*, referred to as “*Paramesorhizobium deserti*” gen. nov. sp. nov. (5). To investigate the underlying mechanisms of antibiotic resistance, we sequenced the genome of strain A-3-E^T^.

The draft genome of strain A-3-E^T^ was sequenced with the HiSeq 2000 platform (Illumina Inc., USA) by using a paired-end strategy. Briefly, a library with the median insert size of 500 bp was constructed, and paired-end sequencing was carried out following the manufacturer’s instructions. The draft genome was *de novo* assembled using SOAPdenovo version 1.06 (6). The coding sequences were predicted using GeneMarkS (7). Putative genes were then annotated by searching against the public databases SwissProt, COG, and KEGG by BLAST (8). The rRNA and tRNA were identified using RNAmmer (9) and tRNAscan-SE version 1.21 (10).

Finally, 880 Mb of raw data were assembled into 41 scaffolds (565 to 1,479,410 bp), defining a draft genome of 5,482,214 bp in length (~160-fold coverage). The G+C content of A-3-E^T^ was determined as 60.93%. A total of 4,946 genes (average length, 960 bp) were predicted, and the total length of the coding sequences is 4,748,013 bp, covering 86.61% of the draft genome. We also identified 47 tRNA genes, with a total length of 3,693 bp, covering 0.067% of the draft genome. In addition, 1 noncoding RNA (ncRNA) was predicted. Bioinformatics analysis identified at least 26 genes encoding potential β-lactamases or proteins with β-lactamases domains in the genome of A-3-E^T^, which deserve further experiments to decipher the mechanisms responsible for their unusual resistance to β-lactam antibiotics.

### Nucleotide sequence accession numbers.

This whole-genome shotgun project has been deposited in GenBank/DDBJ/EMBL under the accession number LNTU00000000. The version described in this paper is the first version, LNTU01000001.
